# Image quality and diagnostic accuracy of inline motion-corrected (moco) first-pass stress myocardial perfusion images

**DOI:** 10.1186/1532-429X-13-S1-O12

**Published:** 2011-02-02

**Authors:** Sujata M Shanbhag, Marcus Y Chen, W Patricia Bandettini, Peter Kellman, H Xue, S Zuehlsdorff, Christopher Glielmi, J Guehring, Li-Yueh Hsu, Andrew E Arai

**Affiliations:** 1 National Institutes of Health, Bethesda, MD, USA; 2 Siemens Corporate Research, Princeton, NJ, USA; 3 Siemens Medical Solutions USA, Chicago, IL, USA

## Introduction

MOCO, a non-rigid image registration algorithm, can minimize myocardial motion and shape changes caused by respiration, arrhythmia, or imperfect gating. A potential benefit of this technique is to enable automated quantitative analysis. On the other hand, MOCO could introduce artifacts and adversely impact diagnostic image quality.

## Purpose

To evaluate image quality and diagnostic performance of MOtion-COrrected (MOCO) first-pass myocardial perfusion images in a referral population.

## Methods

150 consecutive subjects with suspected CAD completed paired vasodilator stress/rest perfusion MRI on a 1.5 Tesla scanner and coronary CT angiogram on a 320-slice scanner. Primary study endpoints are overall MOCO image quality categorized as excellent, good, fair, and poor based on type and extent of image artifacts and the diagnostic accuracy of MOCO stress/rest perfusion images. Both MOCO and raw perfusion images were automatically reconstructed inline for all subjects.

## Results

Of the 150 subjects analyzed, all 150 (100%) MOCO studies had excellent or good overall image quality with diagnostic interpretability. Twelve (8%) MOCO studies had residual artifacts during first pass perfusion. Residual inadequate motion correction due to deep breathing was observed in 8 cases (mild in 7 cases, moderate in 1 case). Residual suboptimal image registration from inadequate cardiac motion compensation from arrhythmia/imperfect gating was seen in 2 cases (mild in 1 case, moderate in 1 case). Both artifacts were seen in 2 cases (mild in both cases).

Diagnostic accuracy of this dataset using the MOCO stress/rest MRI perfusion technique was assessed using invasive angiography adjudicated CTA as the reference standard for CAD. MOCO stress/rest perfusion imaging performed with a sensitivity of 88%, specificity of 96%, positive predictive value (PPV) of 90%, negative predictive value (NPV) of 95%, and accuracy of 94%.

## Conclusions

MOCO image quality was good to excellent in all subjects. MOCO stress/rest perfusion had excellent diagnostic performance using invasive angiography adjudicated CTA as the reference standard for CAD. More importantly, the non-rigid image registration did not introduce clinically significant artifacts.

